# Molecular Details of Polyester Decrystallization via Molecular Simulation

**DOI:** 10.1021/acs.macromol.4c02130

**Published:** 2025-02-07

**Authors:** Daria Lazarenko, Graham P. Schmidt, Michael F. Crowley, Gregg T. Beckham, Brandon C. Knott

**Affiliations:** Renewable Resources and Enabling Sciences Center, National Renewable Energy Laboratory, Golden, Colorado 80401, United States

## Abstract

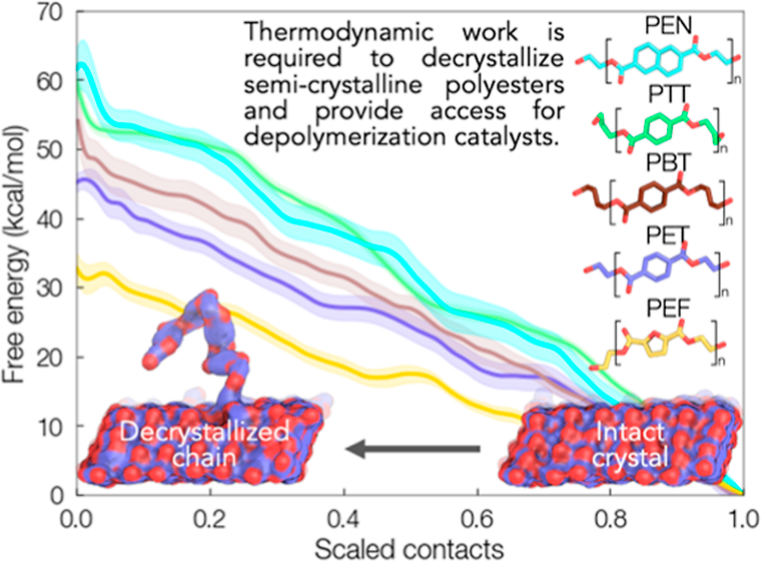

Waste polyesters are a potential feedstock for recycled and upcycled products. These polymers are generally semicrystalline, which presents a challenge for chemical and biological recycling to monomers, and thus the thermodynamic work associated with polyester decrystallization is an important consideration in some depolymerization strategies. Here, we use molecular dynamics simulations to calculate the free energy required to decrystallize a single chain from the crystal surface of five commercially and scientifically important, semiaromatic polyesters (PET, PTT, PBT, PEN, and PEF) in water. Our results indicate the decrystallization work ranges from approximately 15 kcal/mol (PEN) to 8 kcal/mol (PEF) per repeat unit for chains in the middle of a crystal surface. The insight gained into the molecular interactions that form the structural basis of semicrystalline synthetic polyesters can guide the pursuit of more efficient plastic processing, which could include catalyst development, optimizing recycling conditions including pretreatment, enzyme and solvent selections, and design of new materials.

## Introduction

Poly(ethylene terephthalate) (PET) is the world’s most consumed polyester^1^ and constitutes a potentially promising carbon source for recycled and upcycled products.^2^ In many cases, PET deconstruction strategies rely on surface-driven phenomena at a liquid–solid interface. For instance, biocatalytic depolymerization of PET has been demonstrated since 2005,^3,4^ and research efforts in this area have substantially increased since Yoshida et al. reported the hydrolytic degradation and assimilation of PET and its monomers, respectively, by *Ideonella sakaiensis*.^5^ Innovative nonbiological approaches have also demonstrated depolymerization of PET via heterogeneous^6−9^ and homogeneous^9−11^ interfacial catalysis.

PET is generally semicrystalline, with a spatial distribution of amorphous, crystalline, and rigid amorphous regions.^12,13^ While the overall crystalline fraction depends upon processing conditions, PET crystallinity is typically reported between 10% and 40%^14−18^ and can be as high as 50%,^15,19^ a range that enables its wide use in applications requiring structural integrity, high heat resistance, and low permeability (e.g., beverage bottles) and applications where tunable flexibility is necessary (e.g., packaging films) as well as prevalent incorporation into textiles.^14−18,20^ The reported crystallinity of other polyesters also varies significantly, which strongly influences performance, especially mechanical and barrier properties. For example, reported crystallinity for poly(butylene terephthalate) (PBT) and poly(ethylene naphthalate) (PEN) varies from 20 to 50%,^20−24^ while poly(trimethylene terephthalate) (PTT) has a slightly lower range of 15 to 40%.^21^ PTT and PBT exhibit more flexibility and faster crystallization rates than PET, properties that are advantageous for elastic fiber production.^25,26^ PEN is also used in industrial fiber production,^27^ exhibiting favorable UV and chemical resistance, melt temperature, barrier properties, and tensile modulus.^28^ Compared to PET, poly(ethylene furanoate) (PEF) has an order-of-magnitude lower permeability to both oxygen^29,30^ and carbon dioxide,^31^ a 10 °C-higher glass transition temperature,^29^ and can be sourced from biomass;^31,32^ the reported crystallinity range for PEF is comparatively lower and narrower at 10 to 20%.^33,34^ Given their generally high crystalline content, strategies for the complete and efficient deconstruction of polyesters in solid form thus necessitate interfacial catalysis,^35^ and high crystallinity content can be a hindrance, particularly to complete enzymatic deconstruction because the ability of hydrolase enzymes to degrade the crystalline regions is significantly lower than that of the amorphous regions.^19,36−45^

Molecular dynamics (MD) simulations are well-suited to characterize the complex solid-solution interfacial interactions for recalcitrant, semicrystalline polymers. This has been demonstrated in the context of cellulose deconstruction by cellulase enzymes, a scenario with strong analogy to that of PET deconstruction. Therein, MD simulations have provided detailed, quantitative insights, including rates and mechanisms, of the full reaction cycle including chain binding^46−48^ and processivity,^49^ catalysis,^50^ and dissociation^51^ at spatiotemporal scales not readily available experimentally. MD simulations have also enabled a deeper understanding of the molecular roots of biopolymer recalcitrance via detailed understanding of the polymer–solution interface, including decrystallization work^52−54^ and the effect of catalytically induced surface restructuring.^55^ In the context of biologically based deconstruction approaches, high throughput MD simulations have also been leveraged to effectively prescreen for highly active enzymes. For example, Westh and co-workers have developed workflows that exploit linear free energy relationships between properties that are difficult to measure experimentally (e.g., enzymatic activity on semicrystalline polymeric substrate) and those that are estimable via atomistic MD simulation (e.g., substrate binding energy).^56−59^

Here, we have developed crystalline molecular models of five polyesters with either current industrial importance or favorable properties for potential future markets. MD simulations of these models enable estimation of the thermodynamic work required to decrystallize a single chain from the polymer crystal surface in water, and analysis of these trajectories provides insight into the molecular interactions that form the structural basis of semicrystalline synthetic polyesters. The computational methodology presented here can also be considered a framework to quantify the effect on decrystallization of various alternative processing conditions and can inspire the development of targeted deconstruction strategies.

## Computational Methods

**Figure 1:**
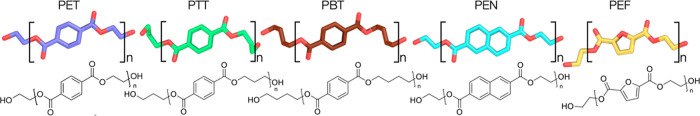
Chemical structures of polyesters under investigation shown as molecular images created in PyMol (top) and ChemDraw (bottom).

The atomic coordinates for the unit cells of PET, PTT, PBT, PEN, and PEF (Figure 1) were downloaded from the Cambridge Structural Database (CSD)^60^ or manually extracted from the original publications presenting the crystal structures.^34,61−64^ Further details, including original references, CSD database identifiers, and unit cell parameters are available in Supporting Information Table S1 and unit cell images are available in Supporting Information Figure S1.

Initial system setup was performed in CHARMM version 44^65^ and subsequent minimization and dynamics were performed with NAMD 2.13.^66^ The CHARMM C36 force field^67,68^ was utilized for the polymers with topologies and additional force field parameters generated via the CHARMM generalized force field (CGenFF) program version 2.5.^69,70^ Further information is given in the Supporting Information Methods. The TIP3P model was utilized for water.^71,72^

In CHARMM, crystals were built with dimensions of 16 chains by 7 chains, and each chain had degree of polymerization (DP) equal to 12 with periodic boundary conditions in all three dimensions. Chain ends were covalently bonded across the periodic boundaries. The “vertical” dimension of 16 chains for a sufficient solvation region to decrystallize a chain into once 12 layers are removed to leave four layers in a subsequent step. A “width” of 7 chains allows for decrystallizing chains while avoiding edge effects as the crystal edges are beyond the nonbond cutoff. A DP of 12 also ensures that the repeat units removed from the crystal surface (once it has been created, vida infra) are beyond the nonbond cutoff distance from the opposite end of the chain at all times.

Following system construction, NAMD was used for minimization and MD simulations. First, a conjugate gradient minimization was performed for 3000 steps on the initial “infinite” crystal. The box volume and atomic positions were then equilibrated for 1 ns at constant pressure (1 atm) and temperature (300 K). During *NPT* dynamics, each dimension of the simulation box was allowed to fluctuate independently. A Langevin thermostat with a collision frequency of 1.0 ps^–1^ was used to maintain temperature at the target of each simulation. Pressure was maintained at 1 atm using a modified Nosé–Hoover method^73^ in which Langevin dynamics are used to control fluctuations in the barostat^74,75^ with a damping time of 100 fs and a period of 200 fs. A nonbonded cutoff distance of 12 Å was utilized, with a switching distance applied between 10 and 12 Å, and a nonbonded pair list distance of 16 Å. The long-range electrostatics were described via the particle mesh Ewald (PME) method^76^ with a sixth-order β-spline and 1 Å grid spacing. The velocity Verlet timestepping integration method was used, with the full nonbonded and electrostatics interactions evaluated on every time step. For all dynamics simulations, a time step of 2 fs was used. The SETTLE algorithm^77^ was used to keep bond lengths to hydrogen atoms fixed for water molecules and SHAKE^78^ for all others.

**Figure 2:**
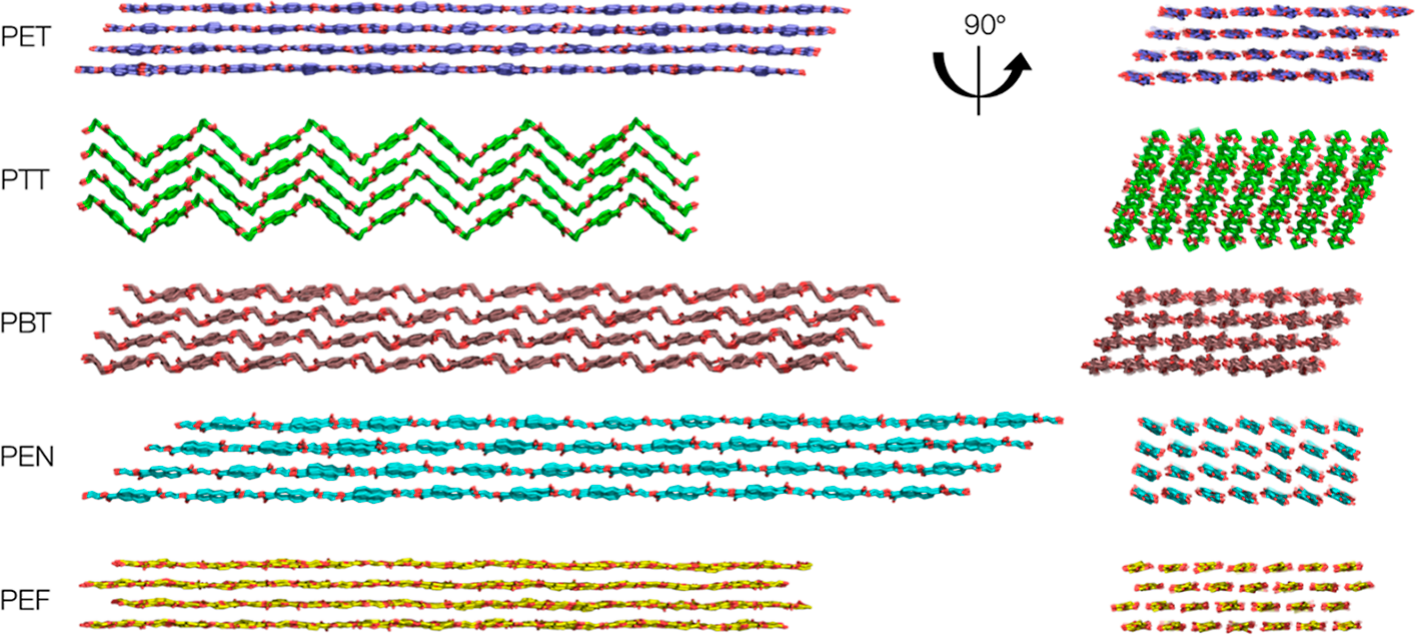
Crystal structures for PET, PTT, PBT, PEN, and PEF. Each polymer chain is 11 repeat units long (DP = 11, horizontal dimension in the images on the left), and each crystal is seven chains wide (left to right in the images on the right). The crystals shown have undergone MD equilibration of 10 ns in water.

Following *NPT* equilibration, chains were slightly shortened (resulting in chains with 11 repeat units) and capped with aliphatic alcohol motifs (Figure 2), and the crystal was trimmed to 7 chains wide by 4 chains deep and solvated in sufficient water to ensure a large solvated gap between each periodic images of the crystal (Supporting Information Figure S2). NAMD was again utilized to perform a conjugate gradient minimization for 3000 steps on the solvated system, followed by 1 ns density equilibration in the *NPT* ensemble at 300 K. Starting with this *NPT* simulation, restraints were placed on the bottom two layers of the crystal. Specifically, a harmonic restraint is applied to the heavy (non-hydrogen) atoms of the bottom two layers with force constants of 4.0 kcal/mol/Å^2^ (bottom layer) and 1.0 kcal/mol/Å^2^ (second layer from bottom); the top two layers of the crystal were completely unrestrained. A 10 ns *NVT* run followed the *NPT* equilibration.

## Results and Discussion

The initial 10 ns *NVT* simulation for each polyester served both as equilibration in preparation for decrystallization simulations and also gave the opportunity to characterize various dynamical traits of the crystalline phase for these polyesters. Root mean square deviation (RMSD, Supporting Information Figure S3) analysis demonstrates the rapid equilibration of the solvated polymer crystals, and general trends from the atom-by-atom root mean squared fluctuations (RMSF, Supporting Information Figure S4) include that, as expected, the mobility of edge chains is greater than that of middle chains and the mobility of chain ends is greater than that of chain interiors. In this regard, PBT displays the largest difference between edge and middle chains, whereas PTT edge chains display similar RMSF to middle chains. All polyesters considered here lack intracrystalline “strong” hydrogen bonds, because the polyesters lack hydrogen atoms bonded to oxygens, with the exception of at the chain ends. However, each polyester participates as a receptor in hydrogen bonds with water molecules (Supporting Information Figure S5a,b), with approximately one strong hydrogen bond per repeat unit for middle chains and 1.5 per repeat unit for edge chains on average. PEF is an outlier to this trend, having considerably fewer hydrogen bonds with water. A significant number of weak hydrogen bonds (defined as O···H–C, oxygen to carbon distance ≤3.5 Å and the O–H–C angle ≤60°) are present, and more prominent differentiation between the different polyesters (Supporting Information Figure S5c–f). Solvent accessible surface area (SASA, Supporting Information Figure S6) can serve as an indicator of polymer accessibility to catalysts, including enzymes. For both middle and edge chains, PEN has the highest accessible surface area to water, though its naphthalene moiety also produces the largest repeat unit of the five polyesters. In contrast, PEF has a smaller aromatic ring, yet has the highest number of weak hydrogen bond interactions for both middle and edge chains.

**Figure 3:**
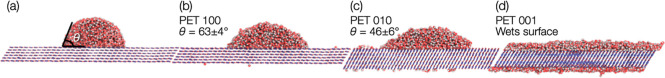
Water contact angle on crystal faces of PET. (a) Definition of the water contact angle, and MD-equilibrated structures for (b) 100, (c) 010, and (d) 001 crystal faces of PET. The starting configuration for each water droplet is an infinite half-cylinder (as shown in (a)) with axis perpendicular to the plane of the page. In panels (a–c), the axis of the PET chains is also into the page; the 001 surface (d) exposes the chain ends (hydroxyl groups), which are in contact with the water. Overlaid on panels (a–c) are the average and standard deviation for water contact angle for the final 50 ns of a 100 ns MD simulation (Supporting Information Figure S7).

Before proceeding with decrystallization simulations, the particular crystalline faces from which to decrystallize a chain need to be identified. We aimed to identify the most hydrophobic face because this is the most likely face for adsorption of hydrolytic enzymes, which have been shown to adsorb with high affinity to the PET surface.^79,80^ The hydrophobicity of polyesters also has a strong impact on processing, performance, and end-of-life recycling strategies. Water contact angle is an efficient method for estimating surface hydrophobicity, which we determined with the following procedure. The water contact angle for PET was examined via MD (Figure 3) on the 100, 010, and 001 crystalline faces, the normal vectors of which are related to the thickness, width, and length of crystallites, respectively.^13,81^ Following the method of Kanduc,^82^ a cylindrical water droplet, infinite along the axis of the droplet, was prepared. This approach involves three phase contact lines whose lengths are independent of droplet size, thus reducing their influence on the contact angle estimate. The details of the analysis of these simulations are described in Supporting Information Figure S7.

The contact angle results (Figure 3) indicated that the 100 face of PET has the highest contact angle of those examined and is thus the most hydrophobic. For subsequent decrystallization, we thus target this face, which is parallel to the aromatic ring of the PET repeat unit, and for consistency, we chose for each of the other polyesters the face whose plane normal is parallel to the aromatic ring normal. For PTT, PBT, PEN, and PEF, these faces are the 100, 100, 100, and 010, respectively (Supporting Information Figure S1). The contact angle estimated here via MD for the 100 face of PET is slightly lower but in reasonable agreement with experimental measurements^83−85^ and computational predictions^86^ for PET water contact angle, which are generally in the range of 72 to 83°. In addition, the finding that the most hydrophobic face for PET is parallel to the plane of the aromatic ring is analogous to the situation for cellulose,^87^ whose hydrophobic 100 face (parallel to the plane of surface glucose rings) has been shown to be the preferential binding face for carbohydrate binding modules.^88−90^

**Figure 4:**
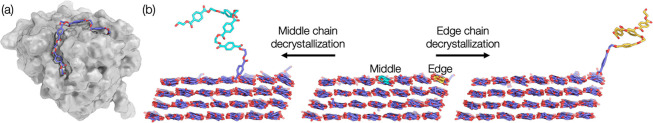
(a) *I. sakaiensis* PETase enzyme with PET tetramer bound, as determined by induced fit docking results.^94,95^ (b) Schematic of the decrystallization of four repeat units from the 100 surface of PET for a middle and edge chain.

The free energy to decrystallize a single chain from the selected hydrophobic face was calculated for each polyester via umbrella sampling in NAMD.^91,92^ The order parameter used to describe the decrystallization process is the fraction of contacts between the extracted chain and the crystal, ranging from 1 (intact crystal) to 0 (complete decrystallization).^52^ The number of contacts was calculated via the NAMD colvars module as the number of heavy atoms in the first four repeat units of the decrystallizing chain with all other heavy atoms in the rest of the crystal, excluding atoms in the rest of the partially decrystallized chain. The cutoff distance for two atoms to be considered in contact was 12.0 Å. This value was chosen because it corresponds to the nonbond cutoff distance such that the four repeat units in the fully decrystallized state have no dispersive interactions with the remaining crystal. The potential of mean force (PMF) could be affected by the choice of the contacts cutoff if it is less than the nonbonded cutoff distance because the decrystallized chain would have dispersive interactions with the surface in that case. Further details on this order parameter are available Supporting Information Figure S8. This choice of four repeat units was inspired by the structure and size of the binding channel of the *I. sakaiensis* PETase enzyme.^5,93,94^ Induced fit docking results have demonstrated that four repeat units of PET can fit into this binding channel (Figure 4a).^94,95^ We also note that decrystallizing four repeat units actually pulls more than four repeat units out of the surface because these four repeat units have to be at least 12 Å away from the surface when the contacts order parameter goes to zero (Figure 4b).

The above procedure was applied to decrystallizing both a middle chain and an edge chain; Figure 4b shows examples for both decrystallization scenarios of four repeat units decrystallizing from the 100 face of PET. Edge chains are expected to be among the easiest to digest while middle chains are likely the most difficult, thus most other chain scenarios within the polymer crystals will likely fall within these limits. As shown in Figure S4, the chain ends are significantly more mobile than the chain interiors, and thus more accessible to solvent (and likely to enzyme active sites) as their position fluctuates into solution. Increased chain flexibility and water accessibility (characteristic of chain ends in our simulations) have been previously correlated with enhanced PET degradation rates by polyester hydrolases.^96,97^ In addition, mass loss of PET samples when incubated with polyester hydrolases is indicative of the release of oligomers or monomers via a degradation mechanism initiated at chain ends.^39^ For these reasons, we focused on decrystallization from chain ends, which also enables direct comparison with prior biopolymer decrystallization results.^52,53^

The initial number of contacts in the fully intact crystal for each polyester was calculated from the unrestrained 10 ns *NVT* simulation described above. The initial configurations for each umbrella sampling window were generated via a subsequent steered MD simulation from this initial number of contacts to zero contacts. During steered MD trajectory, the pulling force constant applied to the collective variable was equal to 0.75 kcal/mol/(contacts)^2^ and the total pulling trajectory length was 30 ns. Atomic coordinates saved every 0.6 ns were used as the initial configurations for the umbrella sampling windows.

During umbrella sampling simulations, a harmonic restraint was applied to heavy atoms on the bottom layer of the polymeric crystal to prevent them from drifting (force constant of 1.0 kcal/mol/Å^2^). We utilized a total of 50 windows, with a biasing potential force constant of 0.0001 kcal/mol/(contacts)^2^. Each umbrella sampling window was simulated for 30 ns total at 300 K in the *NVT* ensemble. Supporting Information Figure S9 presents a representative example of overlapping umbrella sampling histograms for PET. Probability distributions from the final 10 ns were utilized for free energy calculations. The PMF is obtained from the umbrella sampling simulations via the variational free energy perturbation (vFEP) method.^98^ The bootstrapping^99^ method was used for free energy error estimation.

**Figure 5:**
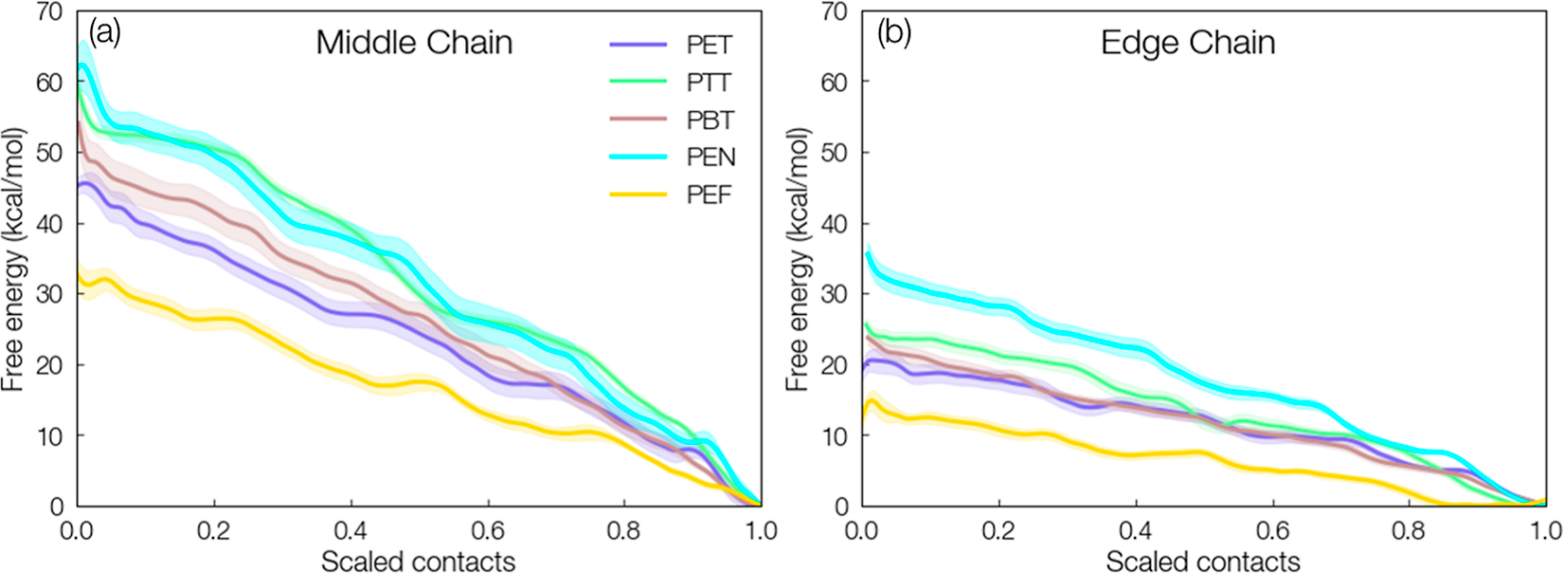
Potential mean force (PMF) as a function of number of atomic contacts for (a) middle and (b) edge chains with the rest of the crystal. PMFs were computed with umbrella sampling, and error bars were estimated via the bootstrapping error analysis technique.

Umbrella sampling simulations were performed for middle and edge chains for each polyester, as illustrated in Figure 4b. For both scenarios, each PMF monotonically increased from the fully intact crystal (scaled contacts equal to 1) to a maximum near zero contacts (i.e., 4 repeat units fully decrystallized) in all systems (Figure 5). For middle chains, the trend in decrystallization work is as follows: PEN (∼62 kcal/mol) > PTT (∼58 kcal/mol) > PBT (∼55 kcal/mol) > PET (∼45 kcal/mol) > PEF (∼32 kcal/mol) (Figure 5a); each value is for decrystallization of four repeat units.

**Figure 6:**
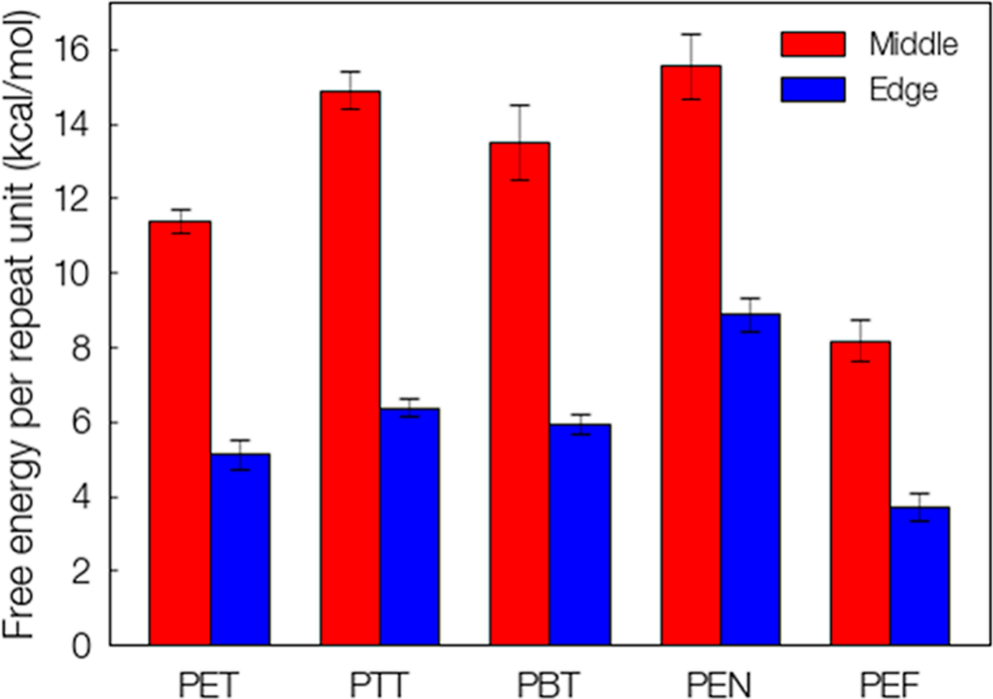
Decrystallization free energy per repeat unit for middle and edge chains. Data for this plot are available in Supporting Information Table S2.

For edge chains (Figure 5b), the PMFs monotonically increase as with the middle chain, but with significantly lower decrystallization work. The observed trend of PEN > PTT > PBT > PET > PEF holds for the edge chain scenario, and the decrystallization work for each is roughly half that of the middle chain case. For both middle and edge chains, four “shoulders” are seen for each system; these represent the stepwise detachment of repeat units from the crystal surface. For middle chains, PEN has the highest decrystallization work (Figure 5a) as well as the highest number of weak intracrystalline hydrogen bonds (Supporting Information Figure S5c). However, PEN also displays the highest decrystallization work for edge chains (Figure 5b), but the least number of weak hydrogen bonds (Supporting Information Figure S5d). This is suggestive that polymer–polymer hydrogen bonds are not the key driver in the recalcitrance of polyesters to decrystallization. In Figure 6, the decrystallization work per repeat unit is computed for each scenario as the maximum in the PMF divided by the number of repeat units that were decrystallized.

Generally, the net change in free energy for the process of decrystallization is due to all thermodynamic changes of all species, i.e. all energetic and entropy contributions including pulling apart a crystal, creating a void in the solvent, as well as new energetic interactions and entropies of water-crystal and water-dissolved polymer. The PMF profiles in this study for each polyester can be correlated to some extent with distinct chemical and physical attributes of the polymers. For example, the naphthalene moieties in the PEN backbone could lead to stronger π–π stacking interactions compared to the single aromatic rings in PET, PTT, and PBT, and may contribute to PEN’s exceptionally high decrystallization work. Naphthalene groups have larger conjugated systems compared to simple benzene rings, and this extended aromatic system can result in stronger interactions with the crystal surface during decrystallization. Also, PBT contains a longer aliphatic chain (butylene) in its backbone compared to PET (ethylene), which may contribute to larger hydrophobic regions that are harder to dissolve in water and thus translate into higher decrystallization free energies. By the same logic, PTT may be expected to fall in between PET and PBT given its three-carbon aliphatic region, yet it shows a decrystallization work that is higher than all studied polyesters except PEN. We surmise that PTT’s high decrystallization work may be related to the physical arrangement of chains within its crystal structure, which displays a unique zigzag pattern (Figure 2) quite different from PET and PBT, both of which display roughly planar chain configurations.

Aromatic stacking interactions is another important factor in the decrystallization work. PEF, with the lowest decrystallization work, contains furan rings in its backbone. A density functional theory (DFT) study calculated energy landscapes for various aromatic heterocyclic molecules with benzene,^100^ and although furan–furan stacking interactions were not calculated, furan’s stacking interaction with benzene was shown to be the weakest interaction of all molecules considered. Benzene has a symmetric, nonpolar structure, whereas furan, with its heteroatom, has slightly weaker stacking interactions due to the influence of its polar nature and the electronic effects of ring oxygen.^100^ PEF also has a unique crystal structure in which chains display furan rings of alternating orientation as well as shifting longitudinally from neighbors in adjacent layers.^34^ We note also that the classical force field utilized here accounts for π–π stacking only implicitly through nonspecific van der Waals forces and electrostatic interactions between aromatic systems. This approximation may not fully capture the directionality and strength variations seen in actual π–π interactions, but the present results, in combination with experimental observations and DFT modeling, can give additional insights into how these interactions may affect polymer decrystallization.

The results for decrystallization work shown in Figures 5 and 6 demonstrate the stability of synthetic polyesters. This can be illustrated by comparison to cellulose, the world’s most abundant biopolymer and itself a recalcitrant semicrystalline polymer.^101^ A previous study estimated the decrystallization free energies for various cellulose polymorphs^52^ and calculated the decrystallization work for a middle chain of cellulose Iβ as 6.7 kcal/mol per cellobiose (the repeat unit of cellulose, a β,1-4 linked glucose dimer). The current study indicates significantly higher decrystallization work for synthetic polyesters. For example, PET exhibits a decrystallization energy of approximately 11.5 kcal/mol per repeat unit for a middle chain, indicating a decrystallization work per repeat unit that is 1.72× higher for PET middle chains as compared to cellulose. This contrast is even more drastic when one considers that the repeat unit of cellulose contains two “ring units” (i.e., glucose) whereas that of PET has only one, indicating a “per ring” decrystallization work that is 3.43× higher for PET than cellulose. Interestingly, the energy needed to decrystallize edge chains for the two polymers is more similar (cellulose Iβ 5.4 kcal/mol per cellobiose; PET 5 kcal/mol per repeat unit). For the polyesters presented here, the free energy required to decrystallize an edge chain is roughly half that for the middle chain case. Unrestrained MD trajectories indicate that edge chains generally exhibit higher RMSF, less frequent polymer–polymer hydrogen bonding, and higher SASA than middle chains (Supporting Information Figures S4, S5c-d, and S6), any or all of which may contribute to their more facile decrystallization. Since the edge chains of the polymeric crystal surface require less free energy to detach than middle chains, they are likely to be detached preferentially, a conclusion also reached for recalcitrant biopolymers.^52,53^ Additionally, when a middle chain is removed, two new edge chains are created, thereby reinforcing the predominance of edge chain removal in the process. A detailed understanding of the surface morphology of polyester crystals, when coupled with the current quantitative results for decrystallization work, would enable the construction of kinetic models describing the full catalytic cycle of polyester hydrolase enzymes.

Our findings on the work required to decrystallize a single polyester chain may help rationalize certain trends in experimentally determined polyester hydrolase activity. PETase enzymes generally have much lower activity on crystalline substrates than amorphous,^36−43,102−104^ and the exceptionally high decrystallization work required for polyesters reported here may provide a rationale for this: perhaps PETases are not able to depolymerize the crystalline regions efficiently because they are unable to decrystallize them. The relative inability of PETase enzymes to decrystallize the crystalline regions has important implications for process choices, notably substrate pretreatment^105,106^ and economics of recycling strategies.^107,108^ Understanding the relative free energies for the full catalytic cycles of PETase enzymes, including decrystallization, is an important consideration in further biocatalyst development.

## Conclusions

In conclusion, this study provides a comparative analysis of the work to decrystallize synthetic polyesters into water, thereby enhancing our understanding of their behavior under conditions that may mimic the early stages of enzymatic and chemical recycling processes under mild conditions. The present study can be conceived of as a framework wherein the effect of alternative processing conditions can be quantified, including the effect of decrystallizing the polymer into an organic solvent, solutions with varying salt content, or even into the active site of an enzyme. Such studies are promising topics for future studies and could spur development of targeted deconstruction strategies, potentially eliminating the need for melt-amorphization before enzymatic depolymerization of PET and other energy-intensive pretreatments for various polymers. Finally, this framework can also be applied to other plastics (e.g., nylons) or may even drive the design of novel materials tailored for more efficient decrystallization and recycling.

## Supporting Information Available

Supplemental methods, figures, and tables including additional background on analysis methods and characterization (PDF)
pet-prm-top.txt (TXT)
ptt-prm-top.txt (TXT)
pbt-prm-top.txt (TXT)
pen-prm-top.txt (TXT)
pef-prm-top.txt (TXT)
