# Supplementary material for: Molecular Details of Polyester Decrystallization via Molecular Simulation
Source: Macromolecules. 2025 Feb 7;58(4):1795–803. doi: 10.1021/acs.macromol.4c02130 (PMC11866931; doi:10.1021/acs.macromol.4c02130)
Supplement: Supplementary file 1 — ma4c02130_si_001.pdf [file ma4c02130_si_001.pdf]

Supplementary Information for:

Molecular details of polyester decrystallization via molecular simulation

*Daria Lazarenko, Graham P. Schmidt, Michael F. Crowley, Gregg T. Beckham, and Brandon C. Knott**

Renewable Resources and Enabling Sciences Center, National Renewable Energy Laboratory; 15013 Denver West Parkway, Golden, Colorado 80401

*Corresponding author. Email: brandon.knott@nrel.gov

Supplementary Methods

Parameter and topology (prmtop) "stream" files are provided in the supplementary materials for the five polyesters included in this study. The coordinates and atoms names were retained from the original publications presenting the atomic coordinates. For PET, PTT, PBT, and PEN, the parameter and charge "penalties" are low (maximum of 5.0 and 0.476, respectively), but those for PEF are substantially higher, with one charge penalty, namely that of the oxygen in the furan ring, and a handful of parameter penalties for dihedrals involving this same oxygen atom exceeding the threshold where additional validation is suggested. Caution should thus be used in interpreting results involving PEF.

Supplementary Figures

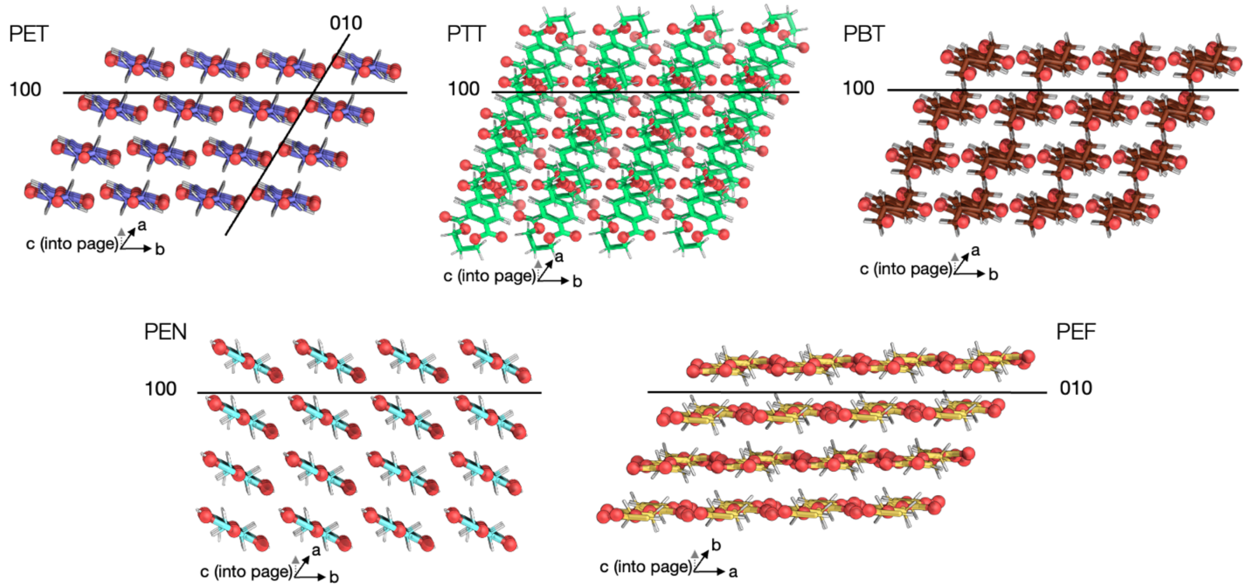

Figure S1. Crystal structure details for PET, PTT, PBT, PEN, and PEF. Several unit cells are shown for each polyester. Axes are defined according to the original publications. For each, the face examined for decrystallization is noted (100 for all except PEF). The 010 face is also noted for PET, for which water contact angle was estimated from MD simulations (main text, **Figure 3**).

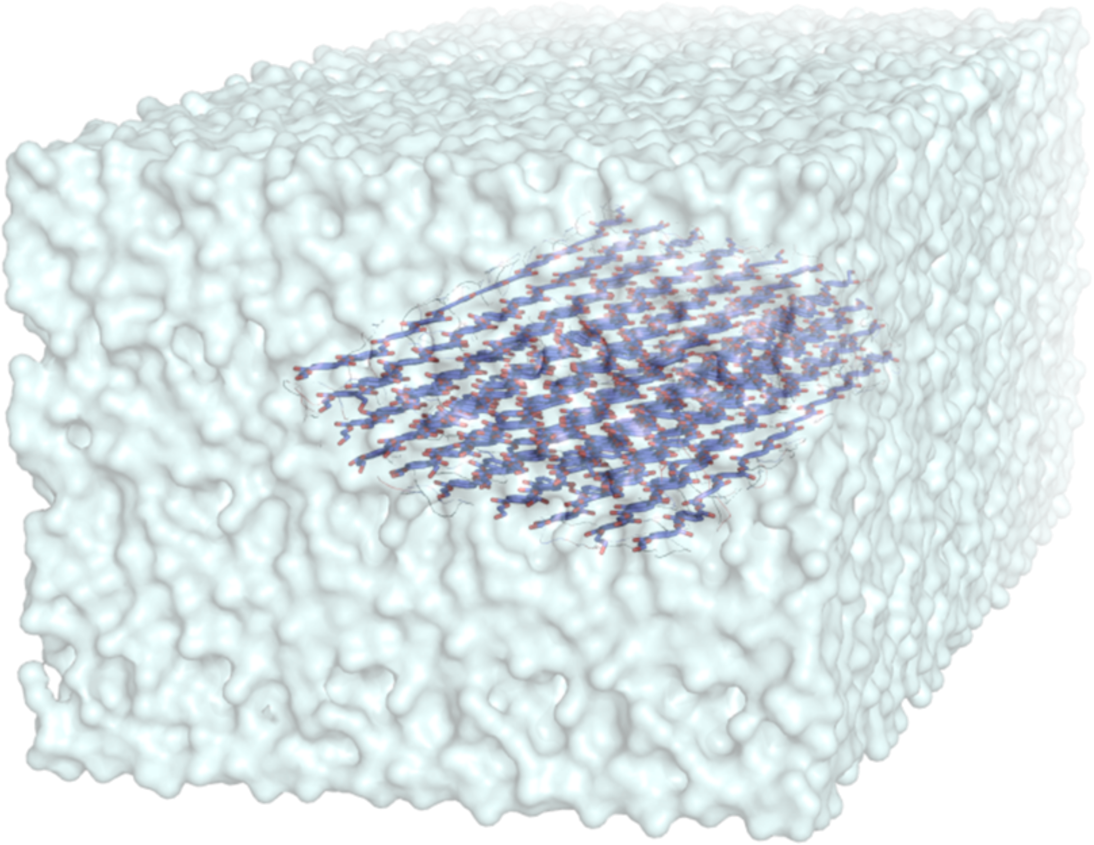

Figure S2. Simulation setup for PET crystalline slab in water before decrystallization.

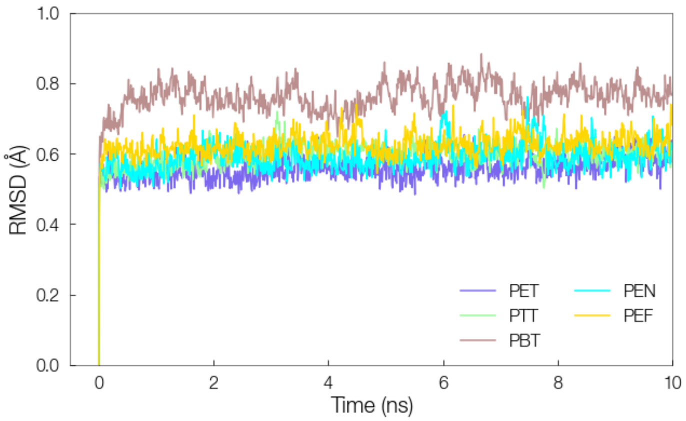

Figure S3. Root-mean-square deviation (RMSD) of polyesters from the original crystal structure. Minor fluctuations and consistent average for all the systems, suggesting that the systems have found a stable conformation throughout the whole simulation. The RMSD calculations were done using python package MDAnalysis.[1, 2]

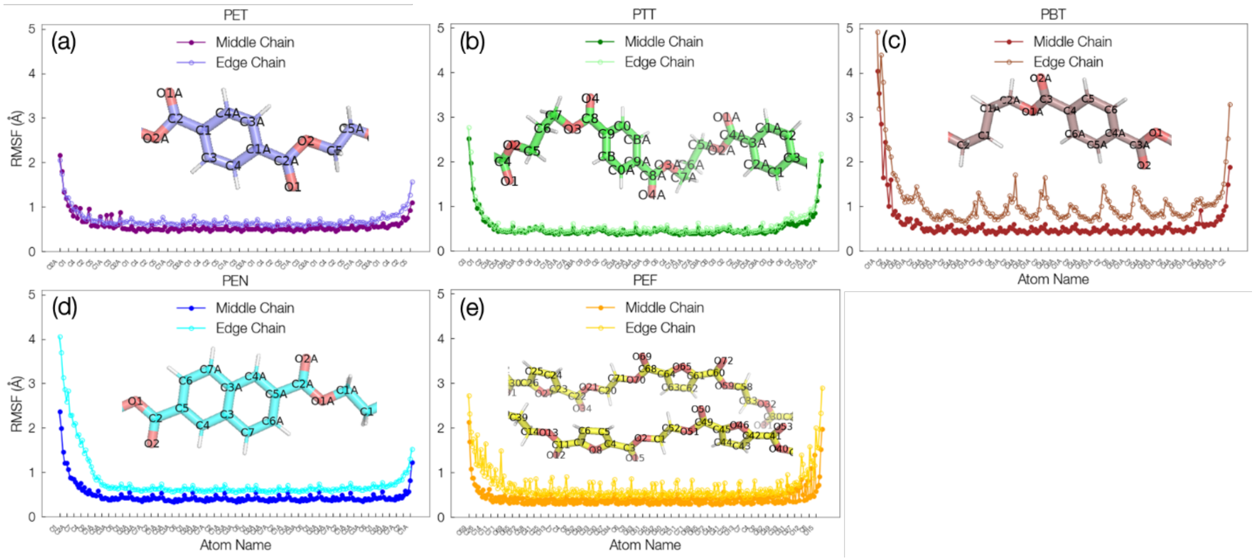

Figure S4. Atom-by-atom root-mean-square fluctuations (RMSF) of five polyesters. To fit the labels on the x-axis, only every fourth atom name was printed. The RMSF calculations were done using python package MDAnalysis.[1, 2]

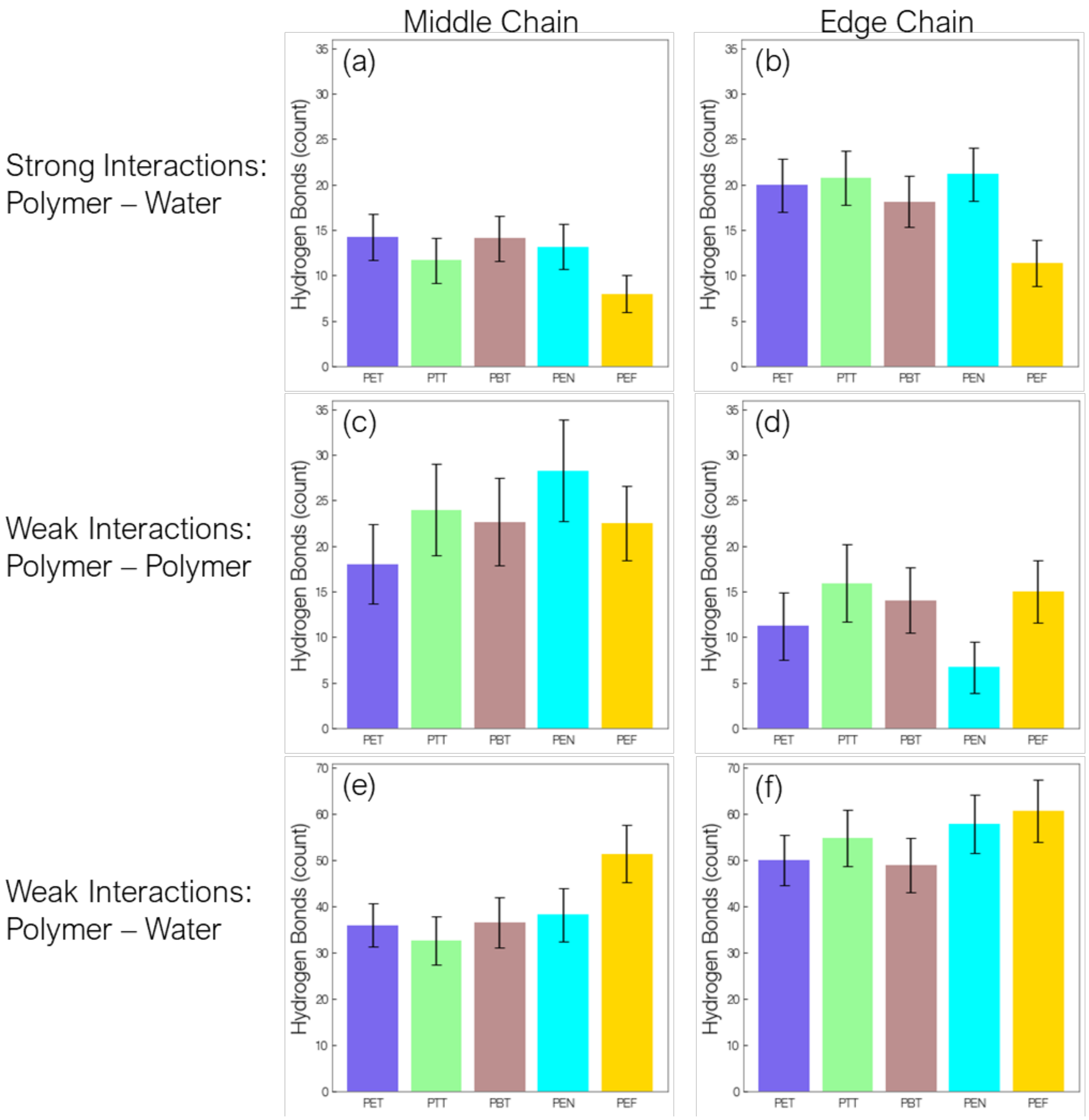

Figure S5. Hydrogen bonds for middle and corner chains for polymer-polymer and polymer-water interactions. Strong hydrogen bonds are defined as O···H-O, with the O-H-O angle ≤ 30° and the O-O distance ≤ 3.5 Å.[3] Weak hydrogen bonds are defined as O···H-C, with the O-H-C angle ≤ 60° and the O-C distance ≤ 3.5 Å.[4-7] The calculation was done in visual molecular dynamics software VMD.[8] Bar heights represent average values, and error bars are calculated as standard deviation.

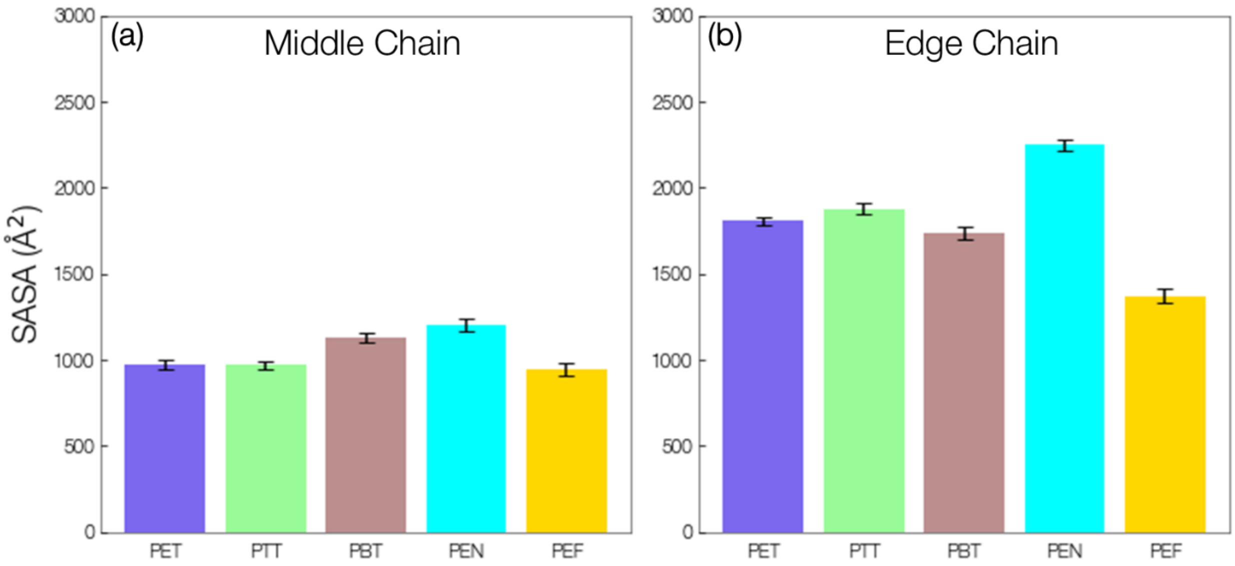

Figure S6. Solvent-accessible surface area (SASA) of middle and edge chain for the five selected polyesters. The calculation was done in visual molecular dynamics software VMD[8] with a probe size of 1.4 Å, consistent with the molecular radius of water. The error bars are calculated as standard deviation.

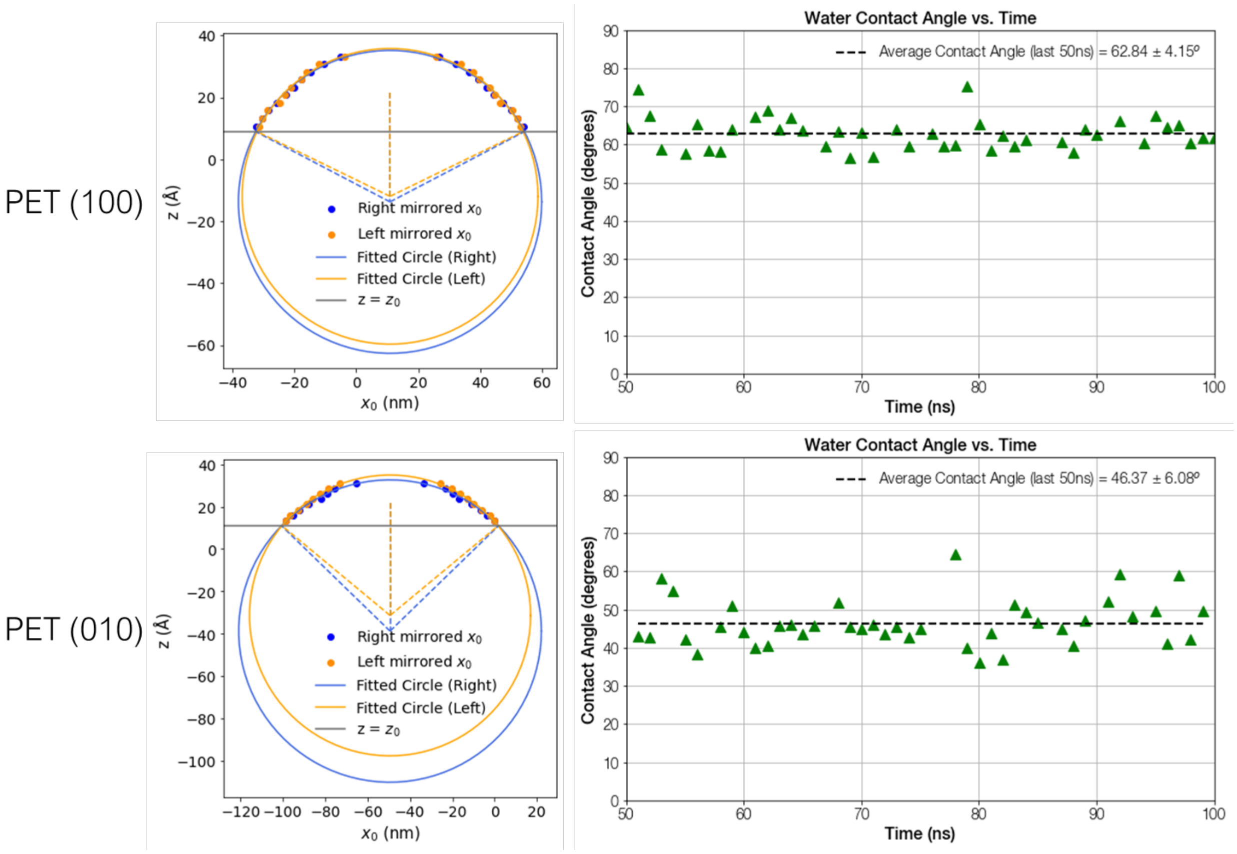

Figure S7. Calculation of water contact angle for 100 and 010 faces of PET. The plots on the left show the fitting of circles to the liquid-vapor interface for the left and right sides of the droplet. The plots on the right display the calculated contact angles over the last 50 ns of simulation. Our analysis follows the approach detailed by Kanduč[9] for cylindrical water droplets using molecular dynamics simulations. Following Kanduč's method, the in-house python code first identifies water molecules in the simulation and calculates center of mass (COM) of the water droplet. We set x-axis along the length of the polymer chains, while the z-axis is perpendicular to the polymeric crystal surface. Then, the code bins the simulation box along the z-axis starting at the solid surface level (z_0) and subsequently along the x-axis from x coordinate of COM of the water droplet to create density profiles for each z-bin. Sigmoidal functions are fitted to these profiles to capture the transition from liquid to vapor phases along left and right side of the droplet around the COM separately. Each point x_0 is determined as the midpoint of a sigmoidal function. These midpoints correspond to the locations where the function's value is halfway between its minimum and maximum. The code uses these x_0 values from different z-bins to fit a circle, capturing the shape of the water droplet's surface. Least squares fitting then determines the parameters of these circles, and tangent lines are drawn to compute contact angles and radii of curvature. The left and right water droplet contact angles are averaged over the last 50 ns of a 100 ns simulation to obtain the final mean value and standard deviation that are reported in **Figure 3** in the main text.

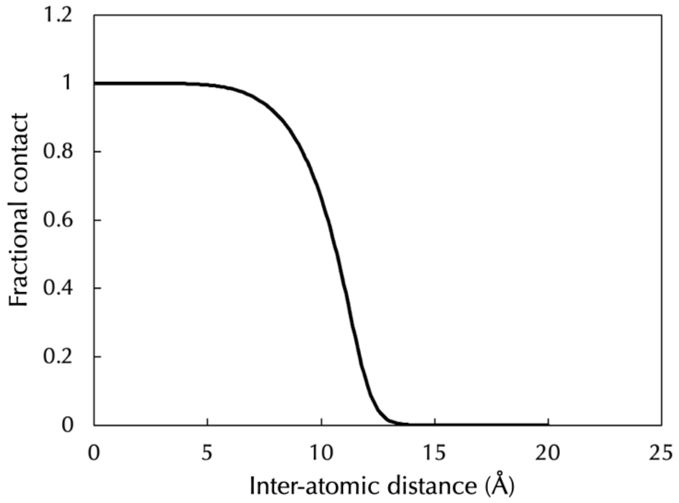

Figure S8. Functional form of the parameter describing the number of contacts. This order parameter is computed via the NAMD[10] coordination number between two groups ("coordnum"), which smoothly goes to zero with the following functional form:

$$C(\text{group1}, \text{group2}) = \sum_{i \in group1} \sum_{j \in group2} \frac{1 - \left(|x_i - x_j|/d_0\right)^n}{1 - \left(|x_i - x_j|/d_0\right)^m}$$

With the parameters used in the present study (d_0=12.0, n=6, and m=48), the functional form shown above is achieved, where d_0 is the cutoff distance, and n and m are exponents that can control the long-range behavior and stiffness.

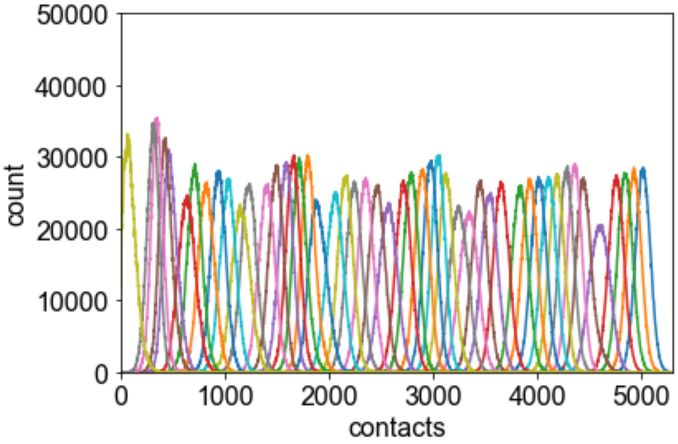

Figure S9. Umbrella sampling histograms for decrystallization of a PET edge chain. A total of 50 windows was sampled with a biasing potential force constant of 0.0001 kcal/mol/(contacts)^2. The neighboring umbrella windows along the reaction coordinates have sufficient overlap, ensuring reliable free energy profiles.

Supplementary Tables

Table S1. Original references, Cambridge Structural Database (CSD) identifiers, and unit cell parameters for polyester crystal structures. For entries without a CSD ID (PTT and PEF), atomic coordinates are taken directly from the original publication.

**Polymer**	**Reference**	**CSD ID**	**a (Å)**	**b (Å)**	**c (Å)**	**α (°)**	**β (°)**	**γ (°)**
PET	Daubeny and Bunn[11]	WIMZEX 01	4.56	5.94	10.75	98.5	118	112
PTT	Desborough *et al.*[12]	n/a	4.6	6.2	18.3	98	90	112
PBT (α)	Yokouchi *et al.*[13]	WINBAW	4.83	5.94	11.59	99.7	115.2	110.8
PEN (α)	Mencik *et al.*[14]	SUSLEX	6.51	5.75	13.2	81.33	144	100
PEF	Mao *et al.*[15]	n/a	5.78	6.78	20.3	90	90	103.25

Table S2. Data for main text Figure 6 for decrystallization free energy per repeat unit for middle and edge chains.

**Polymer / chain**	**Free energy per repeat unit (kcal/mol)**
PET middle	11.39 ± 0.34
PET edge	5.13 ± 0.4
PTT middle	14.90 ± 0.50
PTT edge	6.38 ± 0.24
PBT middle	13.51 ± 0.99
PBT edge	5.92 ± 0.27
PEN middle	15.56 ± 0.87
PEN edge	8.88 ± 0.44
PEF middle	8.18 ± 0.56
PEF edge	3.71 ± 0.37

Supplementary References

(1) Gowers, R. J.; Linke, M.; Barnoud, J.; Reddy, T. J. E.; Melo, M. N.; Seyler, S. L.; Dotson, D. L.; Domanski, J.; Buchoux, S.; Kenney, I. M.; et al. MDAnalysis: A Python Package for the Rapid Analysis of Molecular Dynamics Simulations. In *Proceedings of the 15th Python in Science Conference*, Austin, TX, 2016; Benthall, S., Rostrup, S., Eds.; SciPy: pp 98-105.

(2) Michaud-Agrawal, N.; Denning, E. J.; Woolf, T. B.; Beckstein, O. MDAnalysis: A Toolkit for the Analysis of Molecular Dynamics Simulations. *J. Comput. Chem.* **2011**, *32* (10), 2319-2327.

(3) Luzar, A.; Chandler, D. Hydrogen-bond Kinetics in Liquid Water. *Nature* **1996**, *379* (6560), 55-57.

(4) Steiner, T. Unrolling the Hydrogen Bond Properties of C–H···O Interactions. *Chem. Commun.* **1997**, (8), 727-734.

(5) June Sutor, D. The C–H… O Hydrogen Bond in Crystals. *Nature* **1962**, *195* (4836), 68-69.

(6) Horowitz, S.; Trievel, R. C. Carbon-oxygen Hydrogen Bonding in Biological Structure and Function. *J. Biol. Chem.* **2012**, *287* (50), 41576-41582.

(7) Derewenda, Z. S. C-H Groups as Donors in Hydrogen Bonds: A Historical Overview and Occurrence in Proteins and Nucleic Acids. *Int. J. Mol. Sci.* **2023**, *24* (17), 13165.

(8) Humphrey, W.; Dalke, A.; Schulten, K. VMD: Visual Molecular Dynamics. *J. Mol. Graph.* **1996**, *14* (1), 33-38.

(9) Kanduč, M. Going Beyond the Standard Line Tension: Size-dependent Contact Angles of Water Nanodroplets. *J. Chem. Phys.* **2017**, *147* (17).

(10) Phillips, J. C.; Hardy, D. J.; Maia, J. D. C.; Stone, J. E.; Ribeiro, J. V.; Bernardi, R. C.; Buch, R.; Fiorin, G.; Hénin, J.; Jiang, W.; et al. Scalable Molecular Dynamics on CPU and GPU Architectures with NAMD. *J. Chem. Phys.* **2020**, *153* (4), 044130.

(11) Daubeny, R. d. P.; Bunn, C. W.; Brown, C. J. The Crystal Structure of Polyethylene Terephthalate. *Proc. R. Soc. London, Ser. A Mat.* **1954**, *226*, 531 - 542.

(12) Desborough, I. J.; Hall, I. H.; Neisser, J. Z. The Structure of Poly(trimethylene terephthalate). *Polymer* **1979**, *20* (5), 545-552.

(13) Yokouchi, M.; Sakakibara, Y.; Chatani, Y.; Tadokoro, H.; Tanaka, T.; Yoda, K. Structures of Two Crystalline Forms of Poly(butylene terephthalate) and Reversible Transition between Them by Mechanical Deformation. *Macromolecules* **1976**, *9* (2), 266-273.

(14) Mencik, Z. The Crystal Structure of Poly(tetramethylene terephthalate). *J. Polym. Sci., Polym. Phys. Ed.* **1975**, *13* (11), 2173-2181.

(15) Mao, Y.; Kriegel, R. M.; Bucknall, D. G. The Crystal Structure of Poly(ethylene furanoate). *Polymer* **2016**, *102*, 308-314.
